# Enhancement of magnetization and optical properties of CuFe_2_O_4_/ZnFe_2_O_4_ core/shell nanostructure

**DOI:** 10.1038/s41598-024-57134-7

**Published:** 2024-03-23

**Authors:** A. M. Faramawy, H. M. El-Sayed

**Affiliations:** Department of Physics, Faculty of Science, Ain Shams University, Abbassia, 11566 Cairo Egypt

**Keywords:** Magnetic core shell, Photoluminescence, Superparamagnetic, Magnetic dead layer, AC magnetic loss, Magnetic properties and materials, Nanoparticles

## Abstract

In this work, core/shell of CuFe_2_O_4_/ZnFe_2_O_4_ nanostructure composite was prepared by hydrothermal method. X-ray diffraction (XRD) analysis, transmission electron microscope imaging, energy dispersive X-ray (EDX), and Fourier transform infrared techniques were used to prove the phase formation, morphology, elemental analysis, and cation distribution of core/shell structure, respectively. Furthermore, measurement of the optical properties proved the decrease of photoluminescence (PL) efficiency. The magnetic measurements showed an enhancement of the magnetization by about 63% relative to pure Cu ferrite, and the magnetization curve exhibited superparamagnetic behavior. These results were explained in terms of the depression of the magnetic dead layer thickness in the core/shell structure. The results unleash the promising applications of the prepared samples as transformer cores in the high frequency range and as a photocatalytic agent for water purification and hydrogen production.

## Introduction

Nano ferrites (NFs) have been regarded as crucial materials for modern technologies in the last 20 years. The study of the chemical and physical properties of nano-ferrite (NF) materials is intricate because of the impact of miniaturizing the structure on the magnetic, electric, and optical features that these materials possess^1,2^. In addition, the dopant ions, chemical composition, synthesis procedures, thermal treatments, and shape play a crucial role in determining their characteristics. Applications of NFs include medical, telecommunications, wave absorbers, data recording, chemical sensors, microwave devices, sustainable sources, giant magnetoresistive devices (GMR), magnetic resonance imaging (MRI) technology, drug delivery systems, etc^2–7^.

Intense research is currently in progress on the synthesis and application of magnetic nanoparticles core/shell composites. These important materials have been synthesized and investigated by several research groups during the last few years. There are many reviews in the literature about core/shell ferrite nanoparticles, discussing their different synthesis conditions and techniques^8–11^ and the obtained properties including magnetic^12^, magneto-electric^13^ and optical properties^13^. Their applications in drug delivery^13^, water remediation, and catalysis^14,15^ are also subject to intense research. Zinc ferrite (ZnFe_2_O_4_) is used as semiconductor photo-catalyst for various processes, due to its ability to absorb visible light and its high efficiency, which shows potentially wide applications in photoinduced transformer, photoelectrochemical cells and photochemical hydrogen production^16–20^. Moreover, ZnFe_2_O_4_ as a core and ZnO as a shell were investigated by Rong Shao et al. for photocatalysis and was prepared via solvothermal method^21^. The photocatalytic activity of the novel ZnFe_2_O_4_/ZnO sample was higher than that of pure ZnO and the highest photocatalytic activity was observed in samples prepared with a 1:10 molar ratio of ZnFe_2_O_4_ to ZnO. ZnFe_2_O_4_/SiO_2_/TiO_2_ nanoparticles as magnetic photocatalysts for etodolac degradation was studied by Eryka Mrotek et al.^14^. The coupling between Zn ferrite and oxide materials significantly enhanced etodolac breakdown and mineralization as evaluated by TOC removal. As an alternative to more often utilized metal oxides, spinel copper ferrite (CuFe_2_O_4_) has recently been used for very wide range of applications as a photoanode for water oxidation, photocathodes for hydrogen evolution, photocatalyst and sensor due to its narrow band gap (1.54–1.9 eV)^22,23^. Thu Uyen Tran Thi et al.^24^ synthesized CuFe_2_O_4_/Fe_2_O_3_ core/shell to study the effective photo-Fenton-like catalysts for the methylene blue degradation process using oxalic acid as a radical generator. It was noticed that catalytic performance was two times greater than that of the CuFe_2_O_4_ sample in a 1:2 CuFe_2_O_4_/Fe_2_O_3_, with a rate constant of 2.103 h^−1^ when exposed to UVA light and 0.542 h^−1^ when exposed to visible light. Using laser Raman spectroscopy, Balaji et al.^25^ examined the formation of CuFe_2_O_4_-polyaniline core/shell nanocomposites prepared by in situ polymerization method. Shuo-Hsiu Kuo et al.^26^ fabricated Cu ferrite-polymer as core/shell nanoparticles to study cervical cancer cell photodynamic ablation. It was found that the superparamagnetic characteristics of Cu ferrite-polymer are promising MRI contrast agents and may be utilized as a Fenton catalyst to transform H_2_O_2_ into ROS.

Thus, based on the above survey, there are very few reports on the properties of CuFe_2_O_4_/ZnFe_2_O_4_ core/shell. Herein, hydrothermal technique was used to prepare bare ZnFe_2_O_4_ and CuFe_2_O_4_ nanoparticles, as well as CuFe_2_O_4_/ZnFe_2_O_4_ core/shell nanoparticles for studying the structural, optical, and magnetic properties of these three samples.

## Materials and methods

### Materials

The iron (III) nitrate nonahydrate (Fe(NO_3_)_3_.9H_2_O) (99.99%, Alfa Aesar), copper (II) nitrate hexahydrate (Cu(NO_3_)_2_.6H_2_O) (99.5% LOBA Chemie), zinc (II) nitrate hexahydrate (Zn(NO_3_)_2_.6H_2_O) (98% LOBA Chemie), and sodium hydroxide (NaOH) purchased from Sigma Aldrich utilized in the preparation of the pure and core/shell nano-ferrites were all of analytical grade, and deionized water was used as the solvent.

### Synthesis of copper and zinc ferrites by the hydrothermal method

Copper ferrite nanoparticles were synthesized through the dissolution of (Cu(NO_3_)_2_ · 6H_2_O), and (Fe(NO_3_)_3_ · 9H_2_O) by molar ratio 1:2 in deionized water. The pH of the precursor solution was adjusted to a value of 10 by drop-wise addition of a sodium hydroxide (NaOH) solution while vigorously stirring. After being stirred continuously for one hour, the solution was transferred into a 100 mL Teflon-lined stainless-steel autoclave. The autoclave was subjected to a thermal treatment at a temperature of 180 °C for 18 h. Following the completion of the hydrothermal reaction, the autoclave was subsequently removed from the reaction environment and allowed to cool to ambient temperature. The same process was employed for the preparation of zinc ferrite nanoparticles. The resulting product of both CuFe_2_O_4_ and ZnFe_2_O_4_ was subjected to filtration and subsequent washing with water, repeated multiple times until the pH reached a value of 7.0. After drying at 95 °C for 3 h, the CuFe_2_O_4_ powder was divided into two portions. The first portion was used as bare CuFe_2_O_4,_ and the other portion served as the core for the core/shell composite sample. The chemical reaction for the preparation of both copper ferrite (CoFe_2_O_4_) and zinc ferrite (ZnFe_2_O_4_) nanoparticles is as follows:

1 $${\text{Cu}}\left( {{\text{NO}}_{{3}} } \right)_{{2}} + {\text{2 Fe}}\left( {{\text{NO}}_{{3}} } \right)_{{3}} + {\text{ 8 NaOH}} \xrightarrow {{\Delta \sim 180\,^\circ {\text{C}}}}\, {\text{CuFe}}_{{2}} {\text{O}}_{{4}} + {\text{ 8 NaNO}}_{{3}} + {\text{ 4H}}_{{2}} {\text{O}}$$

2 $${\text{Zn}}\left( {{\text{NO}}_{{3}} } \right)_{{2}} + {\text{2 Fe}}\left( {{\text{NO}}_{{3}} } \right)_{{3}} + {\text{ 8 NaOH}} \xrightarrow{{\Delta \sim 180\,^\circ {\text{C}}}} {\text{ZnFe}}_{{2}} {\text{O}}_{{4}} + {\text{8 NaNO}}_{{3}} + {\text{4H}}_{{2}} {\text{O}}$$

### Synthesis of the CuFe_2_O_4_/ZnFe_2_O_4_ core/shell magnetic nanocomposite

**Figure 1:**
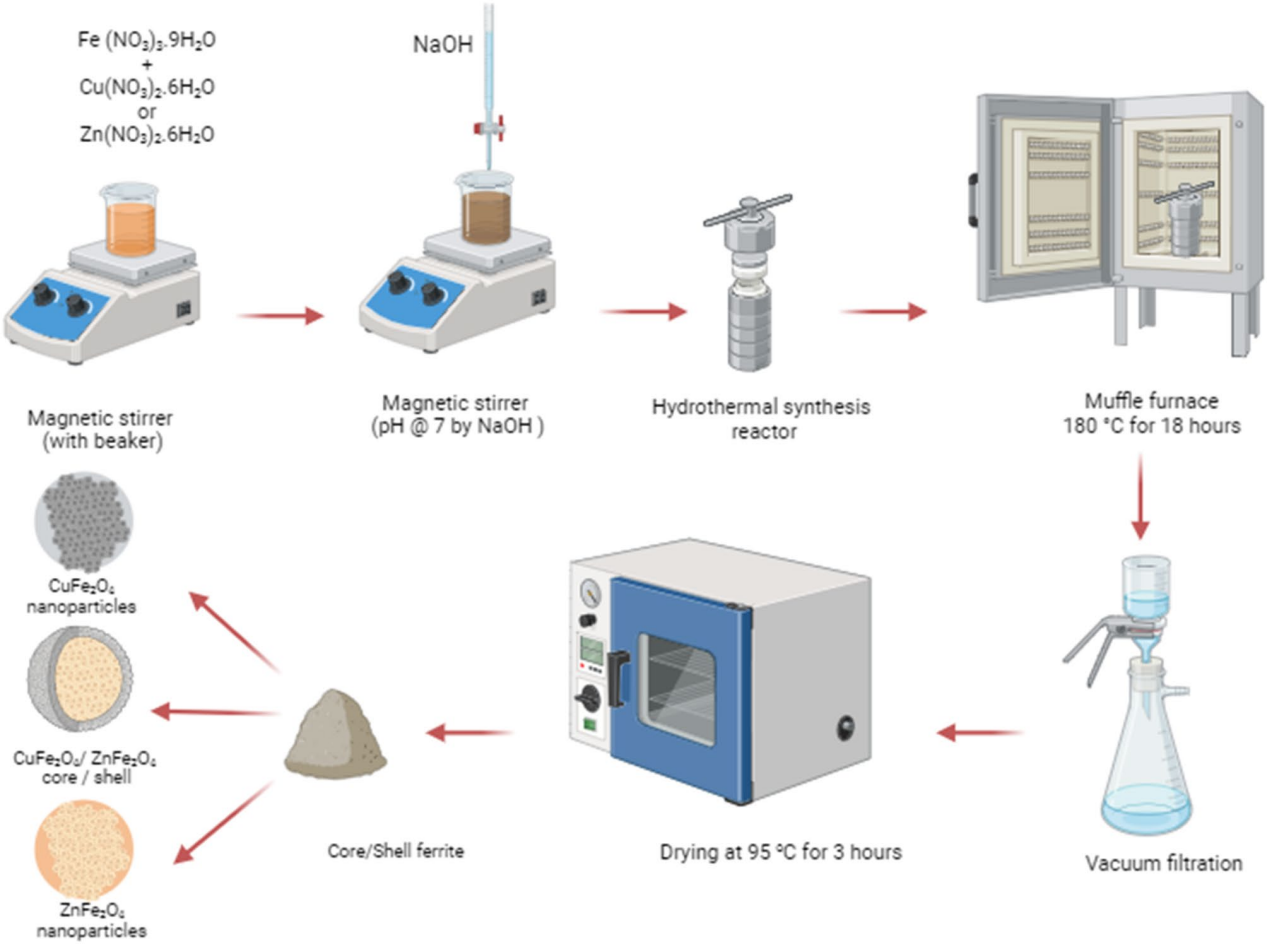
Schematic diagram for the synthesis of the core/shell composite samples by hydrothermal method.

To coat the surface of the prepared CuFe_2_O_4_ (core) with ZnFe_2_O_4_ (as shell), 1 gm of CuFe_2_O_4_ nanoparticles was transferred to the autoclave along with the precursors for the synthesis of ZnFe_2_O_4_. The mass ratio between ZnFe_2_O_4_ to CuFe_2_O_4_ is 2:1. Here, CuFe_2_O_4_ nanoparticles serve as seeds for the crystal growth of ZnFe_2_O_4_ because of the good lattice matching between the core and the shell. The resulting product was subjected to filtration and subsequently washed multiple times with water until the pH of the solution reached 7.0. Finally, the core/shell sample was dried at 95 °C for 3 h. Figure 1 illustrates a schematic diagram for the synthesis of the core/shell composite samples by hydrothermal method.

### Samples characterizations

X-ray diffraction (XRD; BrukerAXSD8Advance, Bruker, Germany) was used to characterize the samples. For studying the microstructure and estimating the particle size, a transmission electron microscope (henceforth, TEM) (Jeol–Jem 1230 electron microscope) was used. Energy Dispersive X-ray (EDX) measurements were carried out using a TESCAN VEGA COMPACT SEM with Tungsten filament as an electron source and an attached EDX detector (Czech Republic). Fourier transform infrared spectroscopy (FTIR) was performed at room temperature in transmission mode (Spectrometer JASCO, 6300, Japan)) in the range of 400–4000 cm^−1^. Room temperature photoluminescence (PL) spectra with an excitation wavelength of 325 nm were obtained using a (FLUOROMAX-2 spectrofluorometer, JOBIN YVON—SPEX, New Jersey; USA). A room-temperature vibrating sample magnetometer (VSM) (model Lake Shore no. 7410 US) was used to measure the magnetic properties in a field up to 20 kG. Finally, the assessment of the AC loss of the samples when exposed to an AC magnetic field with a frequency of 66.5 kHz and a magnetic field strength of 1.45 kAm^-1^; was conducted by measuring the increase in temperature over time.

## Results

### X-ray diffraction (XRD) analysis

**Figure 2:**
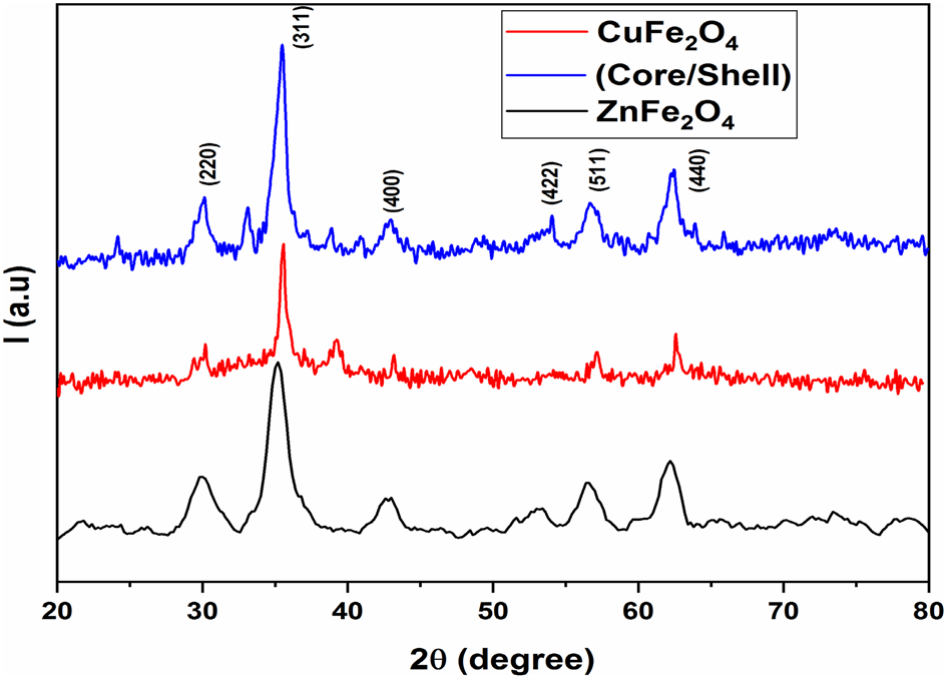
XRD patterns of CuFe_2_O_4_, ZnFe_2_O_4_ and CuFe_2_O_4_/ZnFe_2_O_4_ core/shell samples.

**Figure 3:**
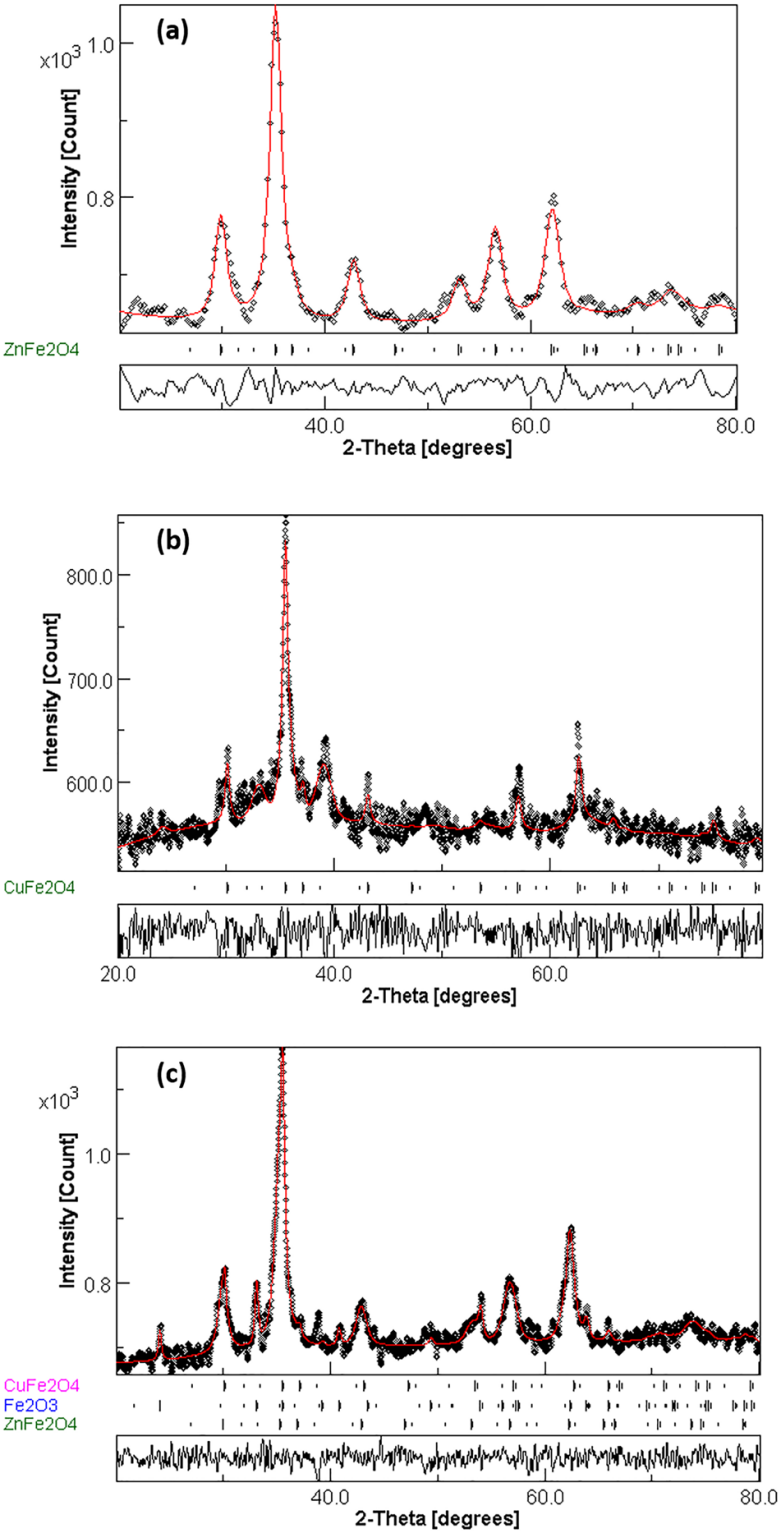
Rietveld refinement profile for (**a**) ZnFe_2_O_4_, (**b**) CuFe_2_O_4_, and (**c**) CuFe_2_O_4_/ZnFe_2_O_4_ core/shell nanoparticles using MAUD program.

Figure 2 shows the X-ray diffraction patterns of Cu ferrite (CuFe_2_O_4_), Zn ferrite (ZnFe_2_O_4_), and CuFe_2_O_4_/ZnFe_2_O_4_ core/shell samples. All of the reflection peaks match well with the typical JCPDS cards No. 34-0425^27^ and 22-1012^28^ of pure CuFe_2_O_4_ and ZnFe_2_O_4_ phases, respectively, with no external peaks indicating that all the produced samples crystallized as a single-phase cubic structure with Fd-3m space group^29^. On the other hand, a small phase percentage of the hematite (α-Fe_2_O_3_) in the core/shell sample was obtained^5,30^. Furthermore, all samples were subjected to Rietveld analysis using the MAUD software programme^31^ in order to accurately characterize the crystal structure and determine the lattice constant ($a$_fit_.) and crystallite size ($L_{fit. }$). Figure 3 illustrates the outcomes of the fitted profile. The process of refinement was carried out iteratively until reaching convergence with a goodness factor approaching one, approximately^2,29^. Table 1 includes both the ($a$_*fit*_*.*) and ($L_{fit. }$) values with the corresponding refinement standard deviation parameter for the investigated samples. It was found from refinement fitting that the phase percentages of ZnFe_2_O_4_, CuFe_2_O_4_, and hematite (α-Fe_2_O_3_) are (82.25%), (2.33%) and (15.42%), respectively. Such a result confirms that the CuFe_2_O_4_ was covered by ZnFe_2_O_4_. The calculated lattice constant ($a$_cal._) for the three samples was computed using the following relation:

3 $$\frac{1}{{d_{hkl}^{2} }} = \frac{{\left( {h^{2} + k^{2} + l^{2} } \right)}}{{a^{2} }}$$

where d_hkl_ denotes the inter-planar spacing in the XRD pattern and (hkl) are the Miller indices of the main peaks. In addition, the Williamson-Hall (W–H) Eq.^32^ is used to obtain the calculated average crystallite size ($L_{cal. }$) as well as the micro strain (ε) for the three samples. The calculated values of ($a$_cal._) and ($L_{cal. }$) are also given in Table 1. One can see that, the lattice parameter values for CuFe_2_O_4_ and ZnFe_2_O_4_ are consistent with those reported in^18,20,27^. In addition to this, the lattice parameter of CuFe_2_O_4_/ZnFe_2_O_4 _core/shell is quite similar to that of pure ZnFe_2_O_4_.The lattice matching between ZnFe_2_O_4_ and CuFe_2_O_4_ causes ZnFe_2_O_4_ crystals to grow as a shell on CuFe_2_O_4_ as a seed^33^.

**Table 1:** Compositions, full width at half-maximum (FWHM), lattice parameter ($a_{cal. } \,\,and\,\,a_{fit.}$), crystallite size ($L_{cal. } \,\,and\,\,L_{fit}$) estimated from XRD, particle size (D) estimated from TEM, refinement standard deviation parameter (σ), strain (**ε**) and magnetization (M_s_) of investigated samples.

Sample	FWHM	$a_{cal.}$(Å)	$a_{fit.}$(Å)	$L_{cal. }$(nm)	$L_{fit. }$(nm)	D (nm)	σ	ε	M_s_ (emu/g)
CuFe_2_O_4_	1.084	8.388	8.398	8.79	8.21	9	0.441	0.0029	7.6
ZnFe_2_O_4_	1.395	8.437	8.440	10.56	9.63	12	0.390	0.0005	10
Core/Shell	0.601	8.418	8.419	19.91	15.79	25	0.372	0.0033	12.4

In addition, when considering the crystallite size of CuFe_2_O_4_/ZnFe_2_O_4_ in comparison to both bare ferrites; it becomes evident that the crystallite size of the core/shell sample is greater than that of bare ZnFe_2_O_4_ and CuFe_2_O_4_ by approximately 9 nm. This observation provides evidence for the formation of core/shell nanoparticles. Furthermore, it can be observed that the lattice strain—Table 1—in the CuFe_2_O_4_/ZnFe_2_O_4_ core/shell is higher compared to that of the pure CuFe_2_O_4_. This increase in lattice strain can be attributed to the minimal difference in lattice parameters between ZnFe_2_O_4_ and CuFe_2_O_4_, providing further evidence for the presence of ZnFe_2_O_4_ as a shell surrounding CuFe_2_O_4_^33^.

### TEM and EDX spectrum

**Figure 4:**
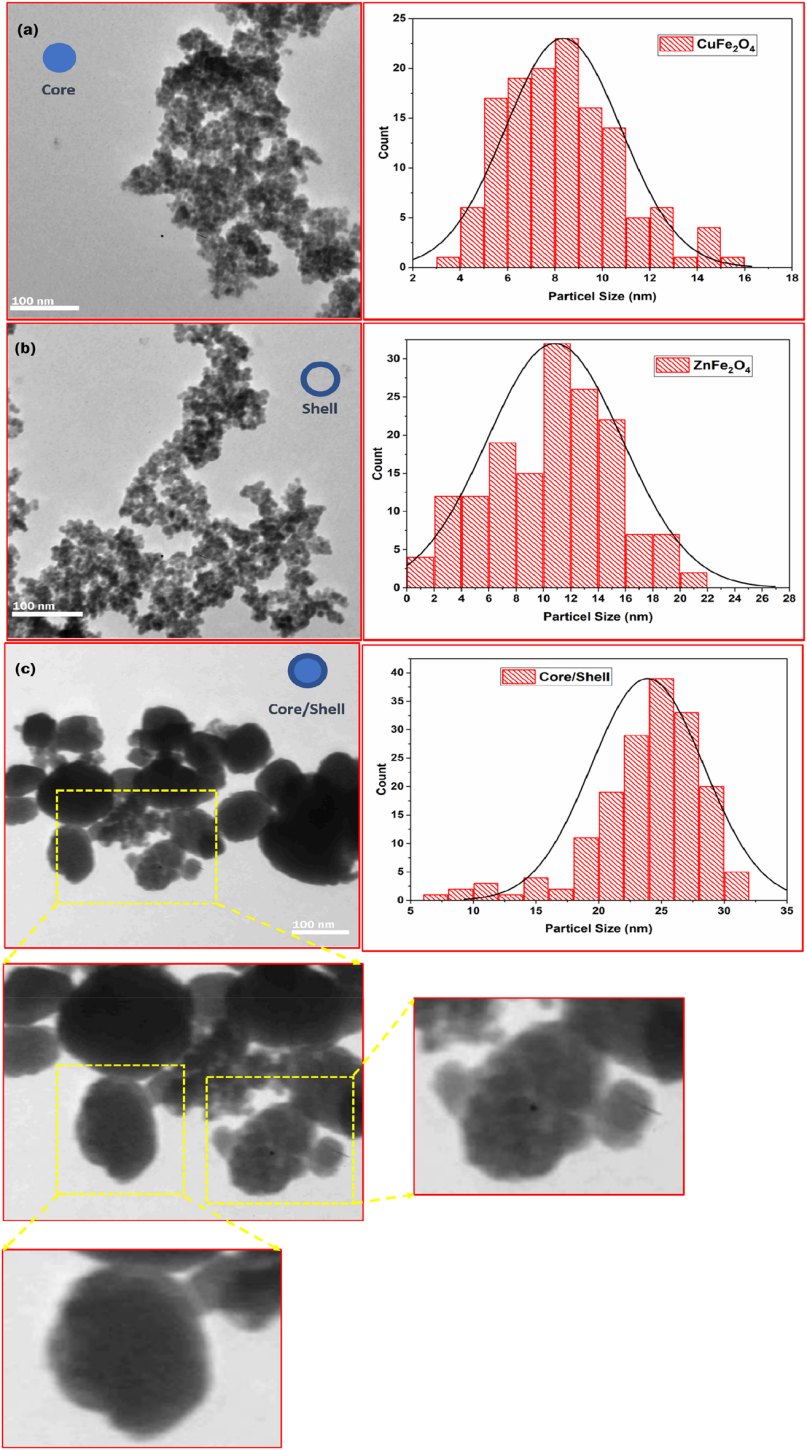
TEM images of (**a**) CuFe_2_O_4_, (**b**) ZnFe_2_O_4_ nanoparticles, and (**c**) CuFe_2_O_4_/ZnFe_2_O_4_ core/shell.

Figure 4 presents transmission electron microscopy (TEM) images depicting the CuFe_2_O_4_, ZnFe_2_O_4_ nanoparticles, and CuFe_2_O_4_/ZnFe_2_O_4_ core/shell. One can see that the shape and particle size distribution of the ferrite particles are uniform. As shown in Fig. 4, the histogram for bare ferrites and core/shell nanoparticles was used to determine the particle size distribution. The particle size was found to be approximately 9, 12 and 25 nm, with an error margin of 2 nm for CuFe_2_O_4_, ZnFe_2_O_4_, and CuFe_2_O_4_/ZnFe_2_O_4_, respectively. These values align well with the measurements obtained from X-ray diffraction (XRD). The uniform shell thickness is around 7 nm thick, while the core size is about 18 nm. However, particle agglomeration occurs as a result of magnetostatic interactions between particles or because nanoparticles experience a permanent magnetic moment proportionate to their volume.

**Figure 5:**
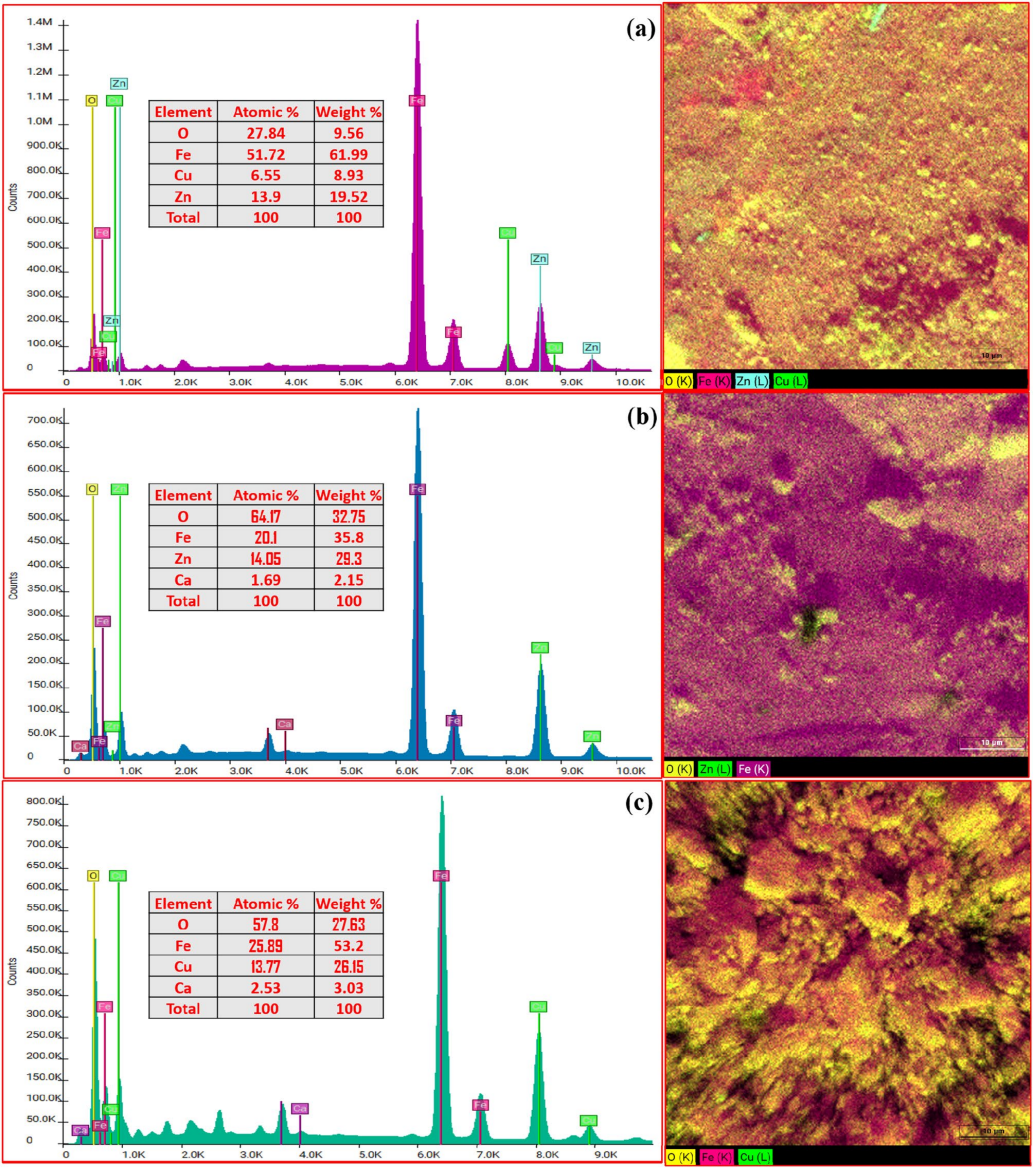
EDX spectra and Mapping of (**a**) CuFe_2_O_4_/ZnFe_2_O_4_ core/shell, (**b**) ZnFe_2_O_4_, and (**c**) CuFe_2_O_4_ nanoparticles.

**Figure 6:**
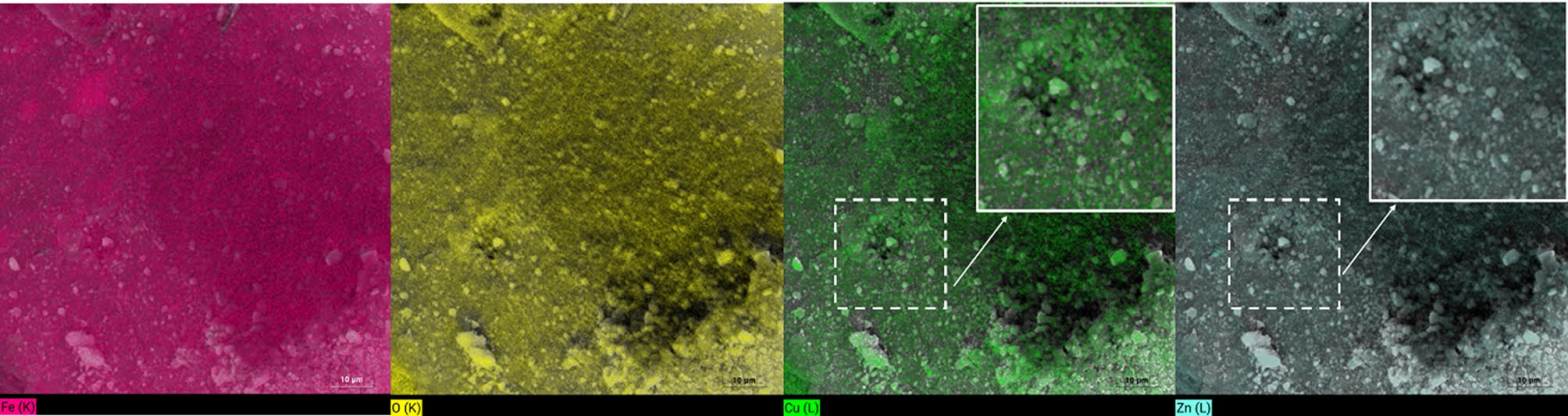
Mapping images of CuFe_2_O_4_/ZnFe_2_O_4_ core/shell nanoparticles.

Furthermore, Fig. 5 illustrates the quantitative EDX analysis conducted at different points and extensive regions to ascertain the elemental composition of the three samples. The results obtained after applying ZAF correction indicated that the atomic weight of the synthesized materials is consistent with the expected stoichiometric ratios. The presence of some Ca peaks in the EDX spectra of both bare ferrites may indicate the presence of small amounts (less than 2.4% by weight) of chemical impurities coming from the precursor materials. Therefore, the samples can be regarded as pure and in strong accordance with the XRD results. From Fig. 6, one can see that at the same point, the Cu and Zn elements, in core/shell sample, have weight percentages of 8.93, and 19.52, respectively, which were confirmed by the XRD refinement analysis. Such a result gives confidence to the formation of core/shell nanoparticles.

### FTIR

**Figure 7:**
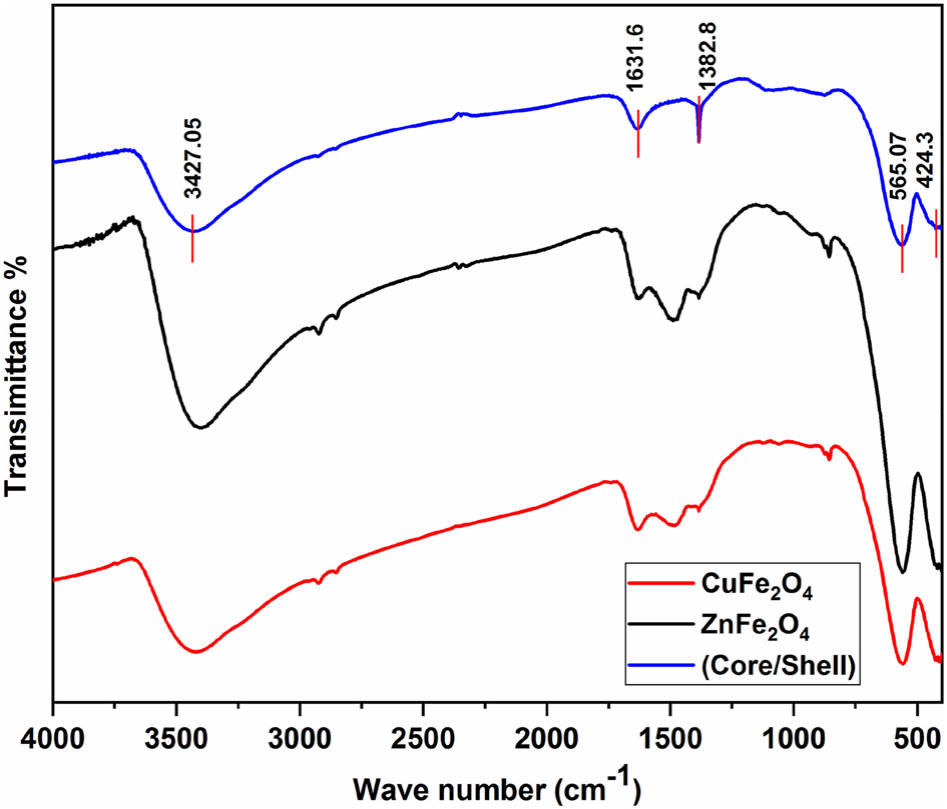
FTIR spectra of CuFe_2_O_4_, ZnFe_2_O_4_ and CuFe_2_O_4_/ZnFe_2_O_4_ core/shell nanoparticles.

Figure 7 displays the Fourier transform infrared (FTIR) spectrum of the investigated samples to confirm the spinel ferrite phase formation and to ensure that there are no chemical residues inside the prepared nanoparticles. The spectrum is presented within the wave-number range of 400–4000 cm^−1^.The infrared spectra of the compound exhibit two prominent absorption bands, namely υ_t_ which are observed at wavenumbers (≈ 560 to 565 cm^−1^) assigned to the tetrahedral complexes and υ_o_ at wavenumbers (≈ 400 to 450 cm^−1^) which associated with the vibrations of high valence cation(s) (such as Fe^3+^–O^2−^) that occupy the octahedral site^34^. The two vibration bands observed in this study are associated with the intrinsic vibrations occurring at the octahedral and tetrahedral sites within the spinel structure, respectively^34^. Both the strong wide band at 3420 cm^−1^ and the weaker band at 1642 cm^−1^ result from the interaction of O–H stretching vibrations with H bonds^35^. Furthermore, the peaks observed at wavenumbers 3427 cm^−1^ and 1632 cm^−1^ are attributed to bending and stretching vibrations of H–O–H which confirms the existence of absorbed water^2,17,19^.

### Photoluminescence (PL) spectroscopy

**Figure 8:**
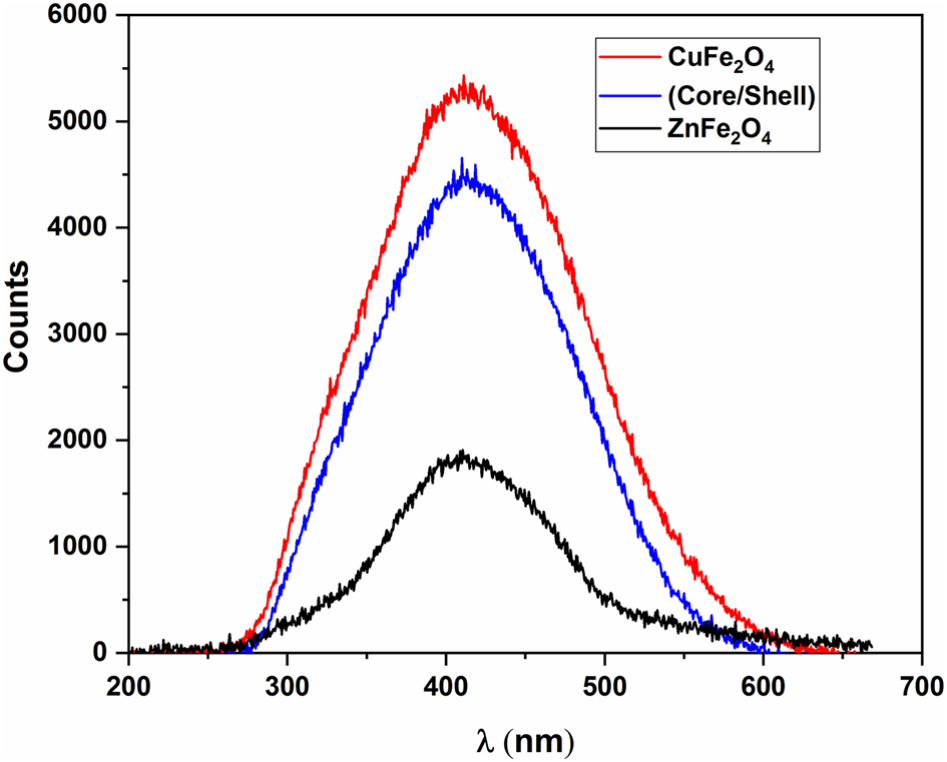
PL spectra for CuFe_2_O_4_, ZnFe_2_O_4_ and CuFe_2_O_4_/ZnFe_2_O_4_ core/shell nanoparticles.

**Figure 9:**
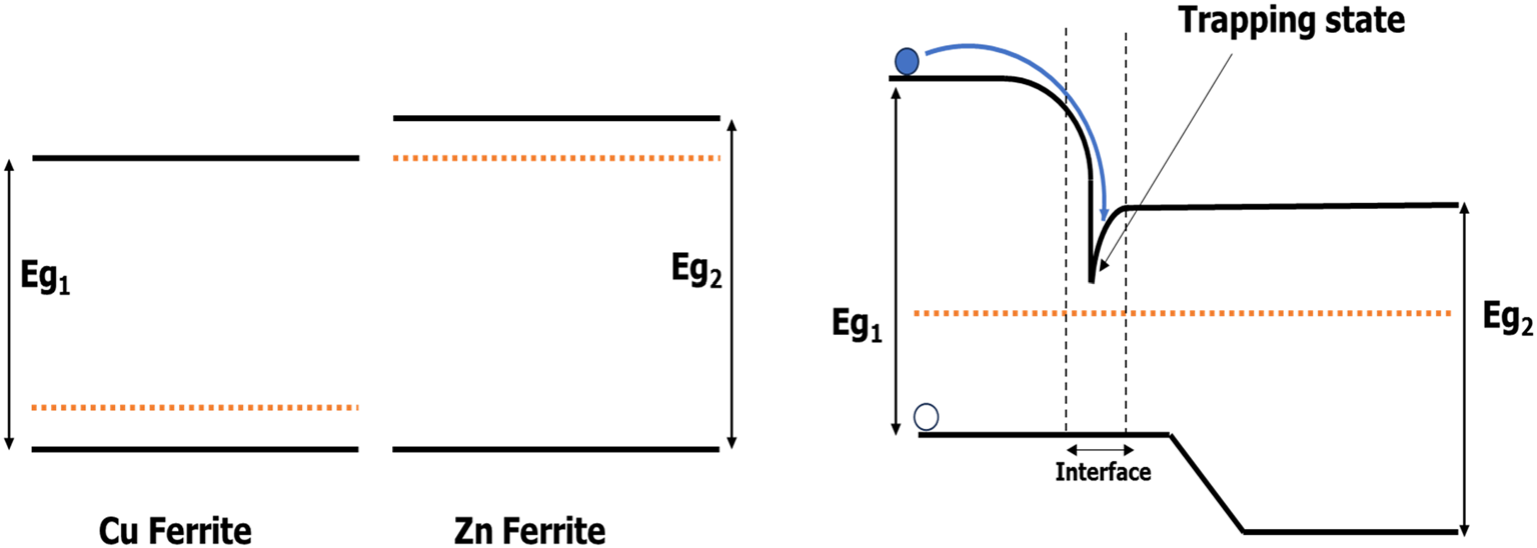
Schematic represents the band diagrams of both ferrites (left) and the trapping state occurs due to the interface between the core and shell (right).

Photoluminescence (PL) is used to study the electronic interaction at the surface between the core and shell structure. Figure 8 shows the PL emission spectra carried out at the excitation wavelength of 325 nm (E_exc._ = 3.8eV), which is greater than the energy gaps of Zn ferrite (2.1 eV)^28^ and Cu ferrite (1.9 eV)^22,23^. One can see that the PL signal is very strong for Cu ferrite while it is very weak for Zn ferrite. This means that the recombination rate between electron–hole pairs is much faster for Cu ferrite than in Zn ferrite. On the other side, the PL signal for core/shell structure is slightly decreased; because of a generation of trapping states at the interface between the core and shell ferrites, as well as it was observed to exhibit a slight redshift, indicating a decrease in wavelength. This could be attributed to the quantum confinement effect^18,28^.The proposed electronic structure is shown in Fig. 9. The modification of PL signal for the core/shell structure could be useful for hydrogen production as well as for water purification as photocatalysis.

### Magnetic properties

**Figure 10:**
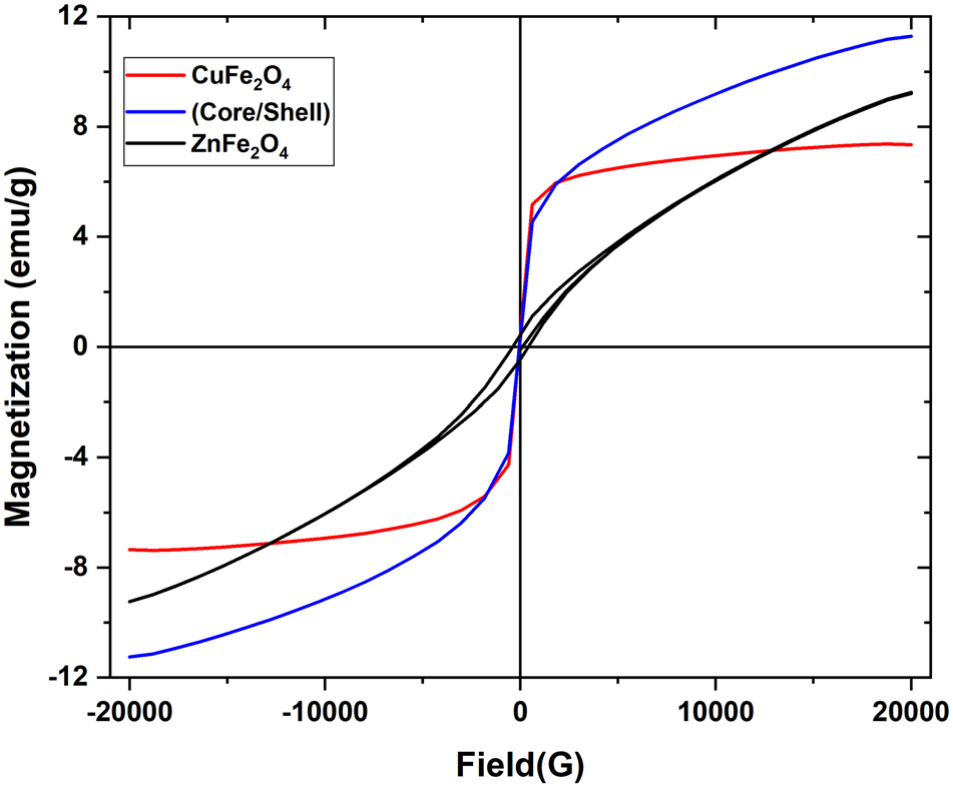
Magnetic hysteresis loop of CuFe_2_O_4_, ZnFe_2_O_4_ and CuFe_2_O_4_/ZnFe_2_O_4_ core/shell nanoparticles.

Figure 10 shows the hysteresis loop for the investigated samples. One can see that, the Cu ferrite (CuFe_2_O_4_) sample has identical ferromagnetic behavior with fast saturation at magnetic field intensity ${ } \ge 5000 G$.

Additionally, the magnetization data extracted from hysteresis loop were fitted employing the Law of approach to saturation (LAS) using the least squares approach, which is demonstrated by the following equation^2,29^.

4 $$M = M_{s} \left( {1 - \frac{B}{{H^{2} }}} \right)$$

**Figure 11:**
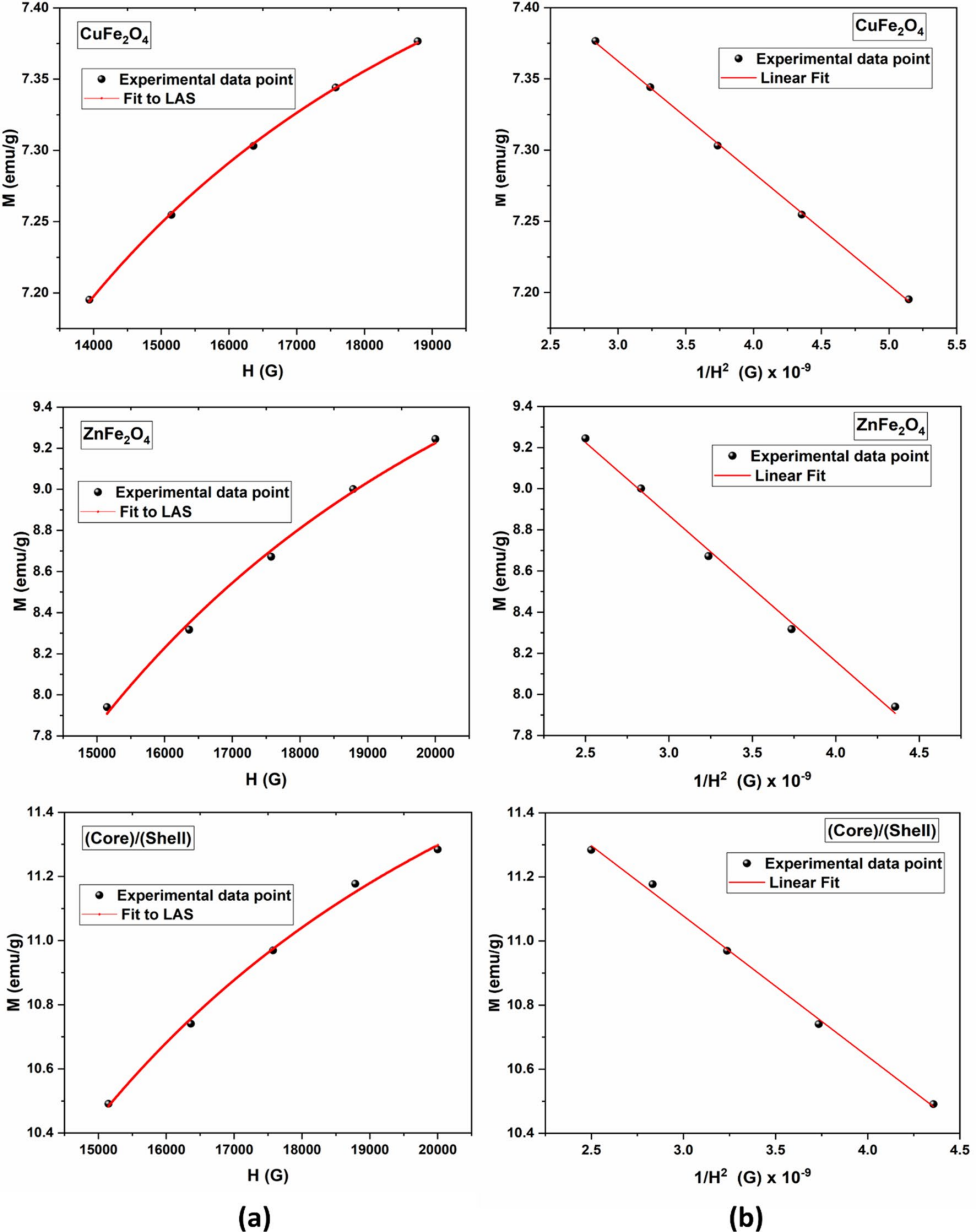
(**a**) ‘Law of approach’ fit to M versus H, and (**b**) linear fit of M versus 1/H^2^ for CuFe_2_O_4_, ZnFe_2_O_4_ and CuFe_2_O_4_/ZnFe_2_O_4_ core/shell nanoparticles.

The parameter (B) is induced by crystal anisotropy, which is related to the cubic anisotropy constant. The above equation describes the relation between the field (H) and (M) in the high field region as illustrated in Fig. 11a. The fitting curves (M vs. 1/H^2^ data) for CuFe_2_O_4_, ZnFe_2_O_4_ and CuFe_2_O_4_/ZnFe_2_O_4_ core/shell nanoparticles are shown in Fig. 11b. The value of the obtained saturated magnetization (M_s_) is about 7.6 emu/g (Table 1), which is lower than the M_s_ value of bulk Cu ferrite (≈ 60 emu/g)^36–38^. This reduction could be explained by the presence of a magnetic dead layer as a result of a high surface-to-volume ratio due to the discontinuity of the magnetic moments at the surface of the nanoparticles and the presence of random canting of particle surface spins at the surface ^39–41^. However, by comparing both values of saturated magnetization, the thickness of the dead layer (δ) could be calculated according to the following relations:

5 $$\left( {{\text{M}}_{{\text{s}}} } \right)_{{{\text{nano}}}} {/}\left( {{\text{M}}_{{\text{s}}} } \right)_{{{\text{bulk}}}} = r_{nano}^{3} { /} r_{bulk}^{3} \,\,{\text{and}}\,\,\delta_{{\text{dead layer}}} = {\text{ r}}_{{{\text{size}}}} {-}{\text{ r}}_{{{\text{eff}}}} \approx 7\,\,{\text{nm}}$$

The Zn ferrites sample exhibits superparamagnetic behavior, and by Langevin function fitting^42,43^, M_s_ was found to be around 10 emu/g which is in agreement with the bulk saturated magnetization value^44,45^, which means there is no magnetic dead layer due to the antiferromagnetic behavior of Zn ferrite. On the other hand, for the CuFe_2_O_4_/ZnFe_2_O_4 _core/shell nanostructure, the saturated magnetization M_s_ increased dramatically up to 12.4 emu/g, which is an interesting behavior. This increasing behavior is not simply a linear combination of the magnetization values of both Cu ferrite and Zn ferrite. Alternately, it can be represented as follows:

6 $${\text{M}}_{{\text{s}}} \left( {\text{core/shell}} \right) = \, 0.{\text{33 M}}_{{\text{s}}} \left( {{\text{ZnFe}}_{{2}} {\text{O}}_{{4}} } \right) \, + \, 0.{\text{67 M}}_{{\text{s}}} \left( {{\text{CuFe}}_{{2}} {\text{O}}_{{4}} } \right) + {\text{M}}_{{\text{s}}} \left( {{\text{Cu}}/{\text{Zn}}} \right)$$

The last term in the above equation is the increase in magnetization due to the interfacial effects between the core and shell. Substituting the experimental values of M_s_ in the above equation, we get:

7 $$\left( {{12}.{\text{4 emu/g}}} \right) = \left( {0.{33}} \right) \, \left( {{1}0{\text{ emu/g}}} \right) + \left( {0.{67}} \right) \, \left( {{7}.{\text{6 emu/g}}} \right) + {\text{M}}_{{\text{s}}} \left( {\text{Cu/Zn}} \right)$$

Then, the value of M_s_(Cu/Zn) = 4.01 emu/g. The enhancement of the magnetization could be explained in terms of the decrease in thickness of the magnetic dead layer due to the continuity of the interaction at the surface of core Cu ferrite and Zn ferrite as a shell. Furthermore, in deep sight, the hysteresis loop of the core/shell sample displays the failure to reach magnetic saturation. This behavior could be explained in terms of the presence of Fe_2_O_3_ as a weakly ferromagnetic or antiferromagnetic phase that initiates the superparamagnetic tendency, i.e., it resists the magnetic moment rotation due to the limited response of the antiferromagnetic moments to the applied field^46^, as well as, it suggests some degree of non-collinear spin structure, which is normally described by a core/shell particle model^47^. It’s valuable to note that the core/shell sample also has superparamagnetic behavior (i.e., H_c_ = 0).

### Magnetic loss (heating ability of the samples)

**Figure 12:**
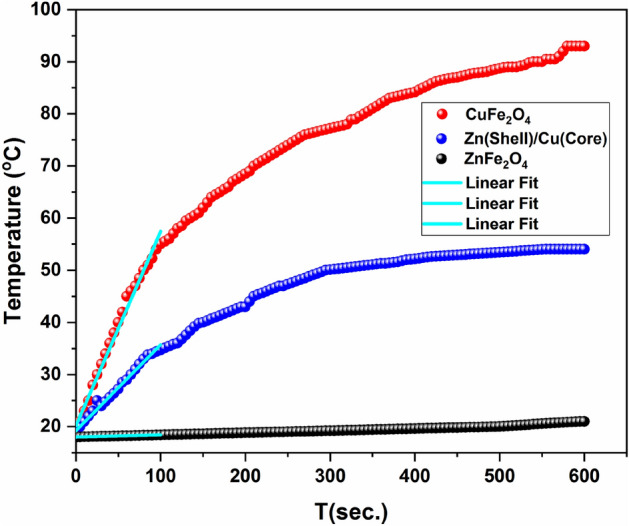
Temperature rise with time for CuFe_2_O_4_, ZnFe_2_O_4_ and CuFe_2_O_4_/ZnFe_2_O_4_ core/shell nanoparticles.

One of the most important studies is the magnetic losses of the material at high frequency. The magnetic loss or heating ability is determined by measuring the increase in temperature over time for the three samples. The design and operation of the used induction heating circuit are described in detail elsewhere^48,49^. Figure 12 depicts the rise in temperature of the investigated samples with time within for an exposure duration of 10 min. It is evident that the Cu ferrite sample has the maximum magnetic loss (the slope of T vs. time) while the Zn ferrite, as expected because of its antiferromagnetic behavior, has the least loss. Typically, hysteresis loss, eddy loss, Néel relaxation, and the Brownian rotation all contribute to the magnetic loss in samples of multi-domain magnetic NPs^29,50^. The generation of hysteresis loss can be attributed to the movements and rotations of magnetic domain walls^51^. When a material undergoes sinusoidal magnetization, the voltage induced within the material exhibits an opposite polarity compared to the voltages responsible for generating the magnetizing current and the alternating magnetic field. Consequently, circular currents are induced, which give rise to magnetic fields that are oriented in the opposite direction to that of the initial current. The eddy current exhibits its maximum strength at the core of the magnetic material particle. Moreover, it is worth noting that hysteresis loss is primarily observed in particles with large crystallites and multiple domains, whereas relaxation loss is observed in particles with a single domain^52^. However, it has been established that the hysteresis and eddy current heating effects can be ignored when dealing with particles with sizes smaller than 20 nm^29^. On the other hand, Néel and Brownian relaxations cause magnetic loss in superparamagnetic materials (i.e., H_c_ = 0). As the sample is in powder form, Brownian relaxation is ruled out because it occurs by particle rotation in the carrier liquid. Hence, for the Cu ferrite sample, we suggested that the primary mechanism responsible magnetic loss is the Néel relaxation^53,54^.

The temperature of the powder samples (CuFe_2_O_4_ and core/shell) increased initially and ultimately reached a state of saturation. The measurement of the heat ability of the samples (ΔT/Δt) is obtained by calculating the slope from the linear segment as shown in Fig. 12. It is valuable to notice that the magnetic loss of core/shell structure decreased in comparison to the bare CuFe_2_O_4_ although its magnetization increased. This property renders the core/shell structure very useful in high frequencies applications. The high magnetization value of the core/shell sample is not reflected on its heating ability. The presence of the Fe_2_O_3_ phase, as previously mentioned in relation to hysteresis measurements, reduces the magnetic loss (the samples' heating ability) by obstructing the rotation of magnetic moments in response to an external magnetic field^55^. This, in turn, reduces the Néel rotation in response to a high-frequency field.

## Conclusions

Core/shell magnetic nanoparticles were synthesized using hydrothermal technique. The mechanism of core/shell formation is determined by the lattice matching between both CuFe_2_O_4_ and ZnFe_2_O_4_ nanoparticles. Moreover, the PL signal of core/shell nanoparticles was found to be between bare ferrites; CuFe_2_O_4_ and ZnFe_2_O_4_. Like change of the PL signal for the core/shell structure compared to both pure ferrite nanoparticles could be advantageous for the creation of hydrogen as well as for the photocatalytic purification of water. The Law of approach to saturation (LAS) was used to deduce the accurate magnetization values for the investigated samples. The magnetization of the core/shell nanocomposite was found to be approximately 63% higher than that of pure Cu ferrite, while the magnetic loss decreased. It is important to acknowledge that the core/shell sample exhibits superparamagnetic behavior. This property holds great promise for various applications.

## Data Availability

The data that support the findings of this study are available from the corresponding author upon request.
